# Sialic Acid Ameliorates Cognitive Deficits by Reducing Amyloid Deposition, Nerve Fiber Production, and Neuronal Apoptosis in a Mice Model of Alzheimer’s Disease

**DOI:** 10.3390/neurosci3010002

**Published:** 2021-12-24

**Authors:** Min Xiao, Chuangyu Yao, Fang Liu, Wei Xiang, Yao Zuo, Kejue Feng, Shuhuan Lu, Li Xiang, Muzi Li, Xiangyu Li, Xiubo Du

**Affiliations:** 1 R&D Center, Cabio Biotech (Wuhan) Co., Ltd., No. 999 Gaoxin Rd., Wuhan 430073, China; min_xiao@cabio.cn (M.X.); fayn_liu@cabio.cn (F.L.); wei_xiang@cabio.cn (W.X.); freya_zuo@cabio.cn (Y.Z.); kejue_feng@cabio.cn (K.F.); sophia_lu@cabio.cn (S.L.); alison_xiang@cabio.cn (L.X.); muzi_li@cabio.cn (M.L.); 2 Guangdong Provincial Key Laboratory for Plant Epigenetics, College of Life Sciences and Oceanography, Shenzhen University, Shenzhen 518060, China; 1900251019@email.szu.edu.cn; 3 Shenzhen Key Laboratory of Marine Biotechnology and Ecology, College of Life Sciences and Oceanography, Shenzhen University, Shenzhen 518060, China

**Keywords:** Alzheimer’s disease, sialic acid, neurodegenerative disease

## Abstract

(1) Background: As a natural carbohydrate, sialic acid (SA) is helpful for brain development, cognitive ability, and the nervous system, but there are few reports about the effect of SA on Alzheimer’s disease (AD). (2) Method: The present study evaluated the effect of SA on cognitive ability, neuronal activity, Aβ formation, and tau hyperphosphorylation in a double transgenic AD (2×Tg-AD) mice model. The 2×Tg-AD mice were randomly divided into four groups: the AD control group, 17 mg/kg SA-treated AD group, 84 mg/kg SA-treated AD group, and 420 mg/kg SA-treated AD group. Mice from all four groups were fed to 7 months of age for the behavioral test and to 9 months of age for the pathological factors investigation. (3) Results: In the Morris water maze, the escape latency significantly decreased on the fifth day in the SA-treated groups. The number of rearing and crossing times in the open field test also increased significantly, compared with the control group. SA treatment significantly reduced amyloid β-peptide (Aβ) and nerve fibers and increased the number of Nissl bodies in the brain of AD mice. (4) Conclusions: SA reduced the neuron damage by reducing Aβ and inhibited tau protein hyperphosphorylation, which improved the cognitive ability and mobility of AD mice.

## 1. Introduction

Alzheimer’s disease (AD) is a progressive neurodegenerative disease with clinical manifestations of cognitive difficulties, reduced language ability, weakened learning and memory abilities, and motor dysfunction [1]. Not only does it cause great harm to the patients themselves, but also brings a heavy burden on the family and society. At present, senile plaques (SP) formed by amyloid β-peptide (Aβ) deposition and nerve fiber tangles (NFT) formed by aggregation of hyperphosphorylated tau protein are recognized as two key pathological factors related to AD [2]. As the aging population increases, so will the number of AD patients in the world. However, so far, there is no effective treatment to prevent or cure AD.

Sialic acid (SA), full name “N-acetylneuraminic acid”, is a natural carbohydrate. As a transmitter of gangliosides and one of the chemical components of the brain, it is located at the end of the glycoprotein side chain of the cell membrane and is an important part of the receptor on the surface of the cell membrane [3,4]. Exogenous free SA can cross the blood–brain barrier (BBB) and enter various tissues [5]. Medical research has shown that SA can improve the learning behavior of animals; help brain development and memory formation in newborns (premature infants); may promote synapse formation; and strengthen the development of the nervous system [6,7]. Thus, SA may have therapeutic potential for AD, but there are few reports about it.

In this study, we aim to explore the effects of sialic acid-rich mixed feeds on learning and memory abilities, anxiety, neuronal activity, and improvement of Aβ formation and tau hyperphosphorylation in a PS1/APP double transgenic AD mice model.

## 2. Materials and Methods

### 2.1. Ethics Statement

This study was conducted in accordance with the Animal Care and Institutional Ethical Guidelines in China. All animal experiments were authorized by the Ethic Committee of Shenzhen University (certificate number: SYXK 2014-0140).

### 2.2. Animals and Treatment

The double transgenic AD (2×Tg-AD) mice, carrying the human gene mutants APPswePSEN1dE9/Nju, and the B6C3F1 WT mice were purchased from GemPharmatech Co., Ltd. (Nanjing, China). Animals were raised in a facility on a 12-h light/12-h dark cycle and at temperature of 22 ± 2 °C with free access to food and water. SPF-grade standard feed and sialic acid mixed feed were provided by CABIO Biotech (No. 999 Gaoxin Rd., Wuhan, Hubei, China.) Co., Ltd. The 3-month-old 2×Tg-AD male mice were randomly divided into four groups: (1) AD control group; (2) 17 mg/kg SA-treated AD group; (3) 84 mg/kg SA-treated AD group; and (4) 420 mg/kg SA-treated AD group. In turn, the age/gender-matched WT mice were divided into two groups: (1) WT control group; and (2) 420 mg/kg SA-treated WT group. There were 12 mice in each group. The AD control group and WT control group were fed with the standard feed. All the mice were fed to 7 months of age for the behavioral test. Feeding continued until 9 months of age (6 months of dosing), where after some mice were taken for liquid nuclear magnetic resonance and functional magnetic resonance experiments; from the remaining mice were taken brain tissue and serum for various biochemical and pathological experiments.

### 2.3. Behavioral Test

#### 2.3.1. Morris Water Maze

A Morris water maze task was carried out, as described previously but with some modifications [8,9], to assess the spatial memory performance of the 9-month-old mice. The water maze was a blue tank (160 cm diameter, 50 cm height) filled to a depth of 26 cm water (21–23 °C). Under this water temperature condition, mice are urgent to find somewhere they can escape from the water because they do not like swimming. The circular water pool was artificially divided into four equal areas—the first to fourth quadrants—placing in advance an invisible platform (12 cm diameter, 1–2 cm below the water surface) in the middle of the third quadrant. During the five consecutive days of training, mice were gently released into the water from the first quadrant to start the test and were allowed up to 1 min to find the submerged platform. If the mice could not mount the platform within 1 min, they would be guided to the platform for 10 s before being returned back to the cage. The escape latency and swimming speed of the mice were recorded. After 24 h and 72 h of the training session, probe trials were performed in which the platform was removed. Each mouse was gently released into the first quadrant and could search freely for 2 min. The time that mice spent in the target quadrant (third quadrant) and the number of mice crossing the previous platform position were recorded. All trials were recorded using a video monitor connected to a computer. The test was run using water maze software (Water Maze MT-200, Chengdu, China).

#### 2.3.2. Open-Field Test

He open-field test equipment consisted of a 1.2 m × 1.2 m square PVC box, and the bottom of it was divided into 25 grids (5 × 5). The test was carried out as described previously but with some modifications [10]. We placed a single mouse in the middle grid of the box and timed it for 3 min, recording the number of times the mouse crosses the grids and the number of rearing (the two front legs were raised at the same time). We wiped off the smell with ethanol before the next mouse experiment to avoid affecting the experimental results.

### 2.4. Tissue and Serum Preparation

The mice were anesthetized with isoflurane and brain tissues were rapidly collected. The left hemisphere was fixed in 4% phosphate-buffered paraformaldehyde, while the right one was dissected into the hippocampus and cortex and kept at −80 °C for the following biochemical analysis. The eyeballs of the mice were removed to collect orbital blood samples, which were stored at 4 °C for 3–4 h, and then centrifuged at 15× rpm for 30 min. The supernatant was aspirated to obtain mice serum.

### 2.5. Serum Biochemical Analysis

Biochemical analysis was performed using a fully automatic biochemical analyzer (iMagic-M7, ICUBIO). The kits were all produced by ICUBIO (Shenzhen, China). All operation steps were performed according to the instructions.

### 2.6. Thioflavin T (ThT) Staining

The brain sections were washed with ddH_2_O several times, then immersed in a 0.001 g/mL ThT working solution (Sigma Aldrich, St. Louis, Mo, USA), and stained at room temperature for 30 min. Subsequently, the sections were washed with ddH_2_O for 5 s and mounted with an anti-fluorescence quenching medium. Finally, the sections were imaged with a confocal microscope (OLYMPUS FV1000).

### 2.7. Silver Staining

After being washed with ddH_2_O several times, the brain sections were treated with formic acid for 5 min and washed three times again. We placed the sections in silver glycine solution (preheated at 37 °C in advance) for 3–5 min, then removed the sections and quickly shook off the residual glycine silver solution on the sections. We put them into reducing solution I (warmed up at 45 °C in advance), and then removed the sections after a few seconds and quickly shook the sections. We placed them in reducing solution II for a few seconds (preheated at 45 °C in advance) and cleaned with ddH_2_O. If the staining background was too deep, it must be treated with sodium thiosulfate solution and washed with ddH_2_O three times. We mounted the slides with anti-fluorescence quenching sealers and imaged them using a confocal microscope (OLYMPUS FV1000).

### 2.8. Nissl Staining

After being washed twice with PBS and dehydrated in graded ethanol, the brain sections were stained with 0.5% cresyl violet for 10 min. We placed these in xylene and mounted them on coverslips. Nissl bodies were visualized by an upright fluorescence microscope (OLYMPUS BX51).

### 2.9. Statistical Analysis

The data were analyzed using GraphPad Prism software and are presented as the mean ± standard error of the mean (SEM). Statistical significance was considered using the following three levels: * *p* < 0.05; ** *p* < 0.01; and *** *p* < 0.001. Statistical analysis was performed using one-way ANOVA followed by Student’s *t*-test.

## 3. Results

### 3.1. SA Treatment Ameliorated the Cognitive Impairment and Depression/Anxiety Behaviors in AD Mice

**Figure 1:**
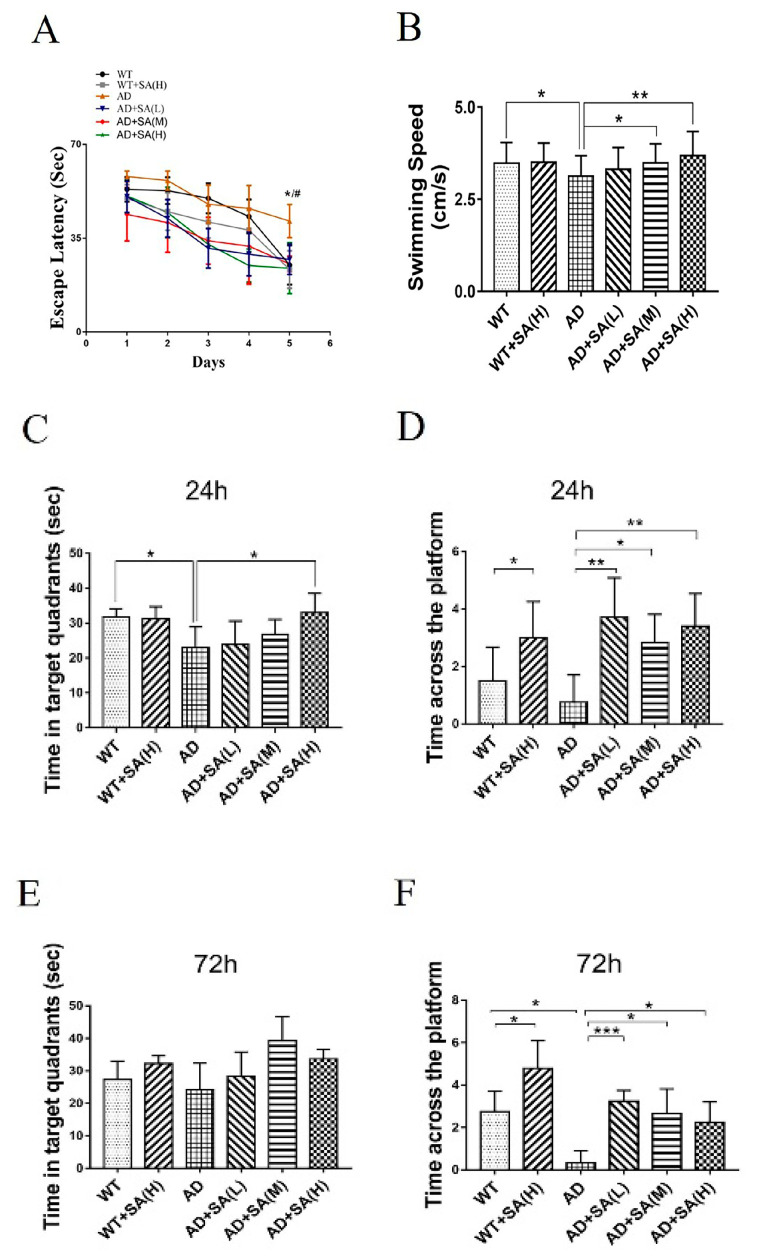
Effect of SA treatment for 4 months on spatial memory and learning ability in 2×Tg AD mice. (**A**) Escape latency. (**B**) Average swimming speed. (**C**) Time in target quadrant 24 h after 5-day training. (**D**) Times crossing platform 24 h after 5-day training. (**E**) Time in target quadrant 72 h after 5-day training. (**F**) Times crossing platform 72 h after 5-day training (# *p* < 0.05, * *p* < 0.05, ** *p* < 0.01, and *** *p* < 0.001). Student’s *t* test was used for statistical analysis. Error bars represent the mean ± SEM (*n* = 12).

The Morris water maze (MWM) was used to evaluate the effect of SA treatment on spatial memory and learning ability in 2×Tg AD mice [9]. After 4 months of SA treatment, all demonstrated groups exhibited progressively shortened escape latency, and AD mice treated with moderate or high SA significantly achieved the shortest time on the fifth day (Figure 1A). The swimming speed of AD mice was significantly lower than that of WT mice, but showed significant improvements when they were treated with moderate or high SA (Figure 1B). It suggested that SA completely recovered the motor- and spatial-learning ability of AD mice. During the probe trial, the target quadrant residence time of AD mice with high SA treatment was significantly higher than that of the AD group after 24 h, and recovered to the state of the WT mice (Figure 1C). The platform-crossing numbers of the SA-treatment groups were significantly higher than that of AD mice, both after 24 h and after 72 h. Similarly, SA also significantly raised the crossing numbers of the WT mice (Figure 1D,F). It indicated that SA obviously improved the short-term and long-term memory not only of AD mice but also of WT mice. All the results showed that after feeding SA for 4 months, not only the motor dysfunction but also the impaired learning and memory of AD mice were rescued, especially at the moderate or high SA concentrations.

**Figure 2:**
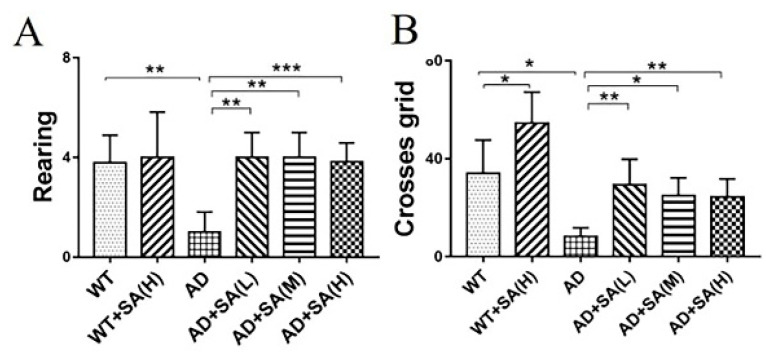
Effect of SA treatment for 4 months on the depression- and anxiety-related behaviors in 2×Tg AD mice. (**A**) Times of rearing. (**B**) Times of crossing grids (* *p* < 0.05, ** *p* < 0.01, and *** *p* < 0.001). Student’s *t* test was used for statistical analysis. Error bars represent the mean ± SEM (*n* = 12).

Furthermore, the open-field test (OFT) was performed to further investigate the effect of SA on the depression- and anxiety-related behaviors of AD mice [10]. After 4 months of SA feeding, compared to WT mice, AD mice demonstrated typical depression/anxiety behaviors, including a significant reduction in the frequencies of grid crossing and rearing (Figure 2A,B). Compared to AD mice, the SA-treatment groups remarkably improved the depression/anxiety behaviors, as indicated by significantly increased grid crossings and rearing frequencies of AD mice (Figure 2A,B). The above behavioral MWM and OFT tests (Figure 1 and Figure 2) suggested that the cognitive deterioration and the depression/anxiety behaviors of AD mice was effectively improved by SA treatment, especially at moderate or high SA concentrations.

### 3.2. SA Significantly Suppressed the Pathological Hallmarks of AD Mice

**Figure 3:**
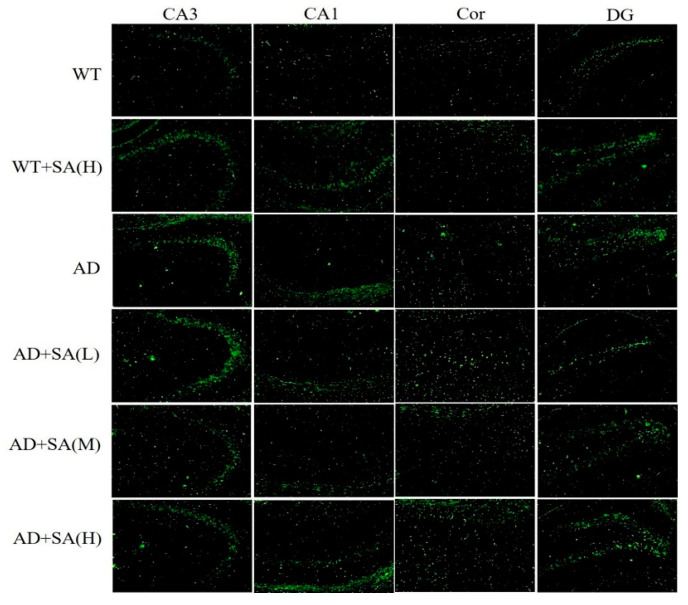
Effect of SA treatment on deposition of Aβ in the hippocampus and cortex of 2×Tg AD mice (10×). CA3:CA3 area of the mouse hippocampus; CA1: CA1 area of the mouse hippocampus; Cor: cortical area; DG: dentate gyrus area of the hippocampus (*n* = 4; scale bars = 50 μm).

Many studies have shown that the interaction between Aβ and tau may be central to the development of AD [11,12], and APP/PS1 transgenic mice develop age-related accumulation of plaques and tangles in brain [13]. Detection of plaques in the hippocampus and cortex of the mouse brain by thioflavin T staining, to some extent, reflects the accumulation and distribution of Aβ in the brain. According to the results, the areas occupied by the Aβ plaques of WT mice were all reduced significantly in both the cortex and hippocampus, compared with the AD mice, but they had increased plaques after being fed SA. In CA3 and DG, although the low SA treatment was negligent in reducing plaques of AD mice, the plaques of AD mice treated with moderate or high SA were less than that of AD mice. Compared with AD mice, low or moderate SA treatments reduced Aβ plaques in CA1 of AD mice, while little effect was observed in AD mice treated with high concentration of SA. Only moderate SA treatment reduced Aβ in the cortex (Figure 3). All these results demonstrated that a moderate SA concentration had a significant effect on reducing Aβ aggregation in AD mice.

**Figure 4:**
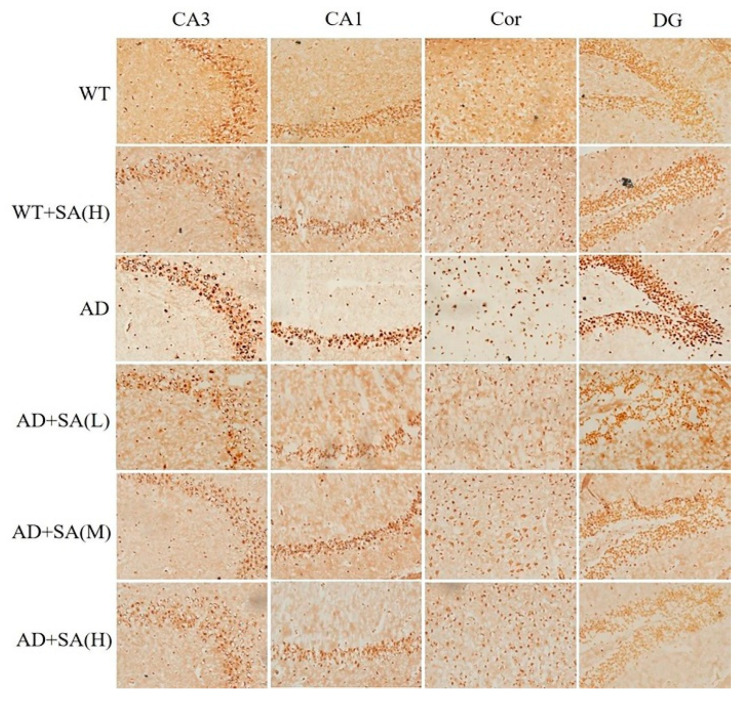
Effect of SA treatment on neurofibrillary tangles in the hippocampus and cortex of 2×Tg AD mice (20×) (*n* = 4; scale bars = 25 μm).

Glycine silver staining was used to detect the neurofibrillary tangles (NFTs) in the hippocampus and cortex of the mouse brain. The formation of NFT was due to the excessive phosphorylation of tau protein, which caused it to entangle with each other after falling off the microtubule tangle of nerve fibers [14,15]. From the results, the NFT of WT mice was significantly less than that of the AD group in CA3, CA1, DG, and Cor, and the SA treatment made the differences more pronounced. The NFT in AD mice with SA treatment was less than that of AD mice. There was a negative correlation between the NFT and SA concentration in each brain region of the AD mice (Figure 4).

### 3.3. SA Reversed Neuronal Loss in AD Mice in a Concentration-Independent Manner

**Figure 5:**
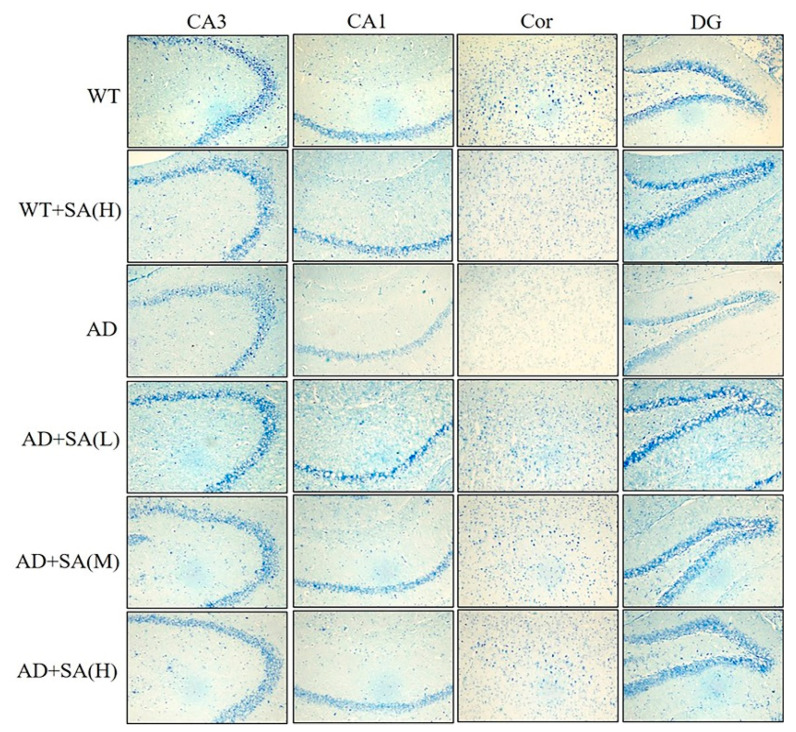
Effect of SA treatment on neuronal activity in the hippocampus and cortex of 2×Tg AD mice (4×) (*n* = 4; scale bars = 50 μm).

The number of Nissl bodies is inversely proportional to the loss of neurons, and the Nissl staining was used to detect the number of Nissl bodies on the rough endoplasmic reticulum in the brain tissue of mice [16]. As shown in Figure 5, the number of Nissl bodies in CA3, CA1, DG, and Cor of AD mice was significantly less than that of WT mice, and Nissl bodies in the WT mice treated with high SA was significantly less than that in WT mice in the cortex area. Significant increases in the Nissl bodies’ number and density in all the mentioned regions were observed in AD mice treated with low SA, compared with AD mice. Feeding moderate or high SA also slightly raised the Nissl bodies in AD mice. It revealed that SA may reduce the loss of neuronal activity in AD mice, but it was not concentration dependent.

### 3.4. SA Lowered Blood Lipids and May Prevent Vascular Diseases

**Figure 6:**
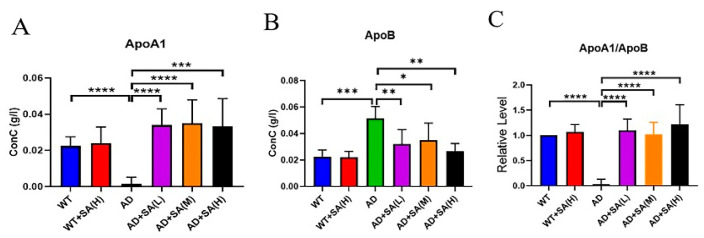
Effect of SA treatment on blood lipid-related indicators in 2×Tg AD mice. (**A**) Apolipoprotein A1. (**B**) Apolipoprotein B. (**C**) Apolipoprotein A1 is better than apolipoprotein B (* *p* < 0.05, ** *p* < 0.01, *** *p* < 0.001, and **** *p* < 0.0001). Student’s *t* test was used for statistical analysis. Error bars represent the mean ± SEM (*n* = 6).

Apolipoprotein A1 (ApoA1) and apolipoprotein B (ApoB) are lipid-related indicators [17,18]. ApoA1 comes from high-density lipoprotein, whose main role is to transport lipids in tissues and organs to the liver, and the liver decomposes the lipids [19]. ApoA1 has a negative correlation with the incidence. ApoB exists on the surface of low-density lipoprotein and exist to recognize and take up low-density lipoprotein, increasing the incidence even at low levels [18]. From the results, SA extremely and significantly increased the level of ApoA1 in AD mice, even reaching normal levels (Figure 6A). The level of ApoB in AD mice was extremely and significantly higher than that of WT mice and AD mice with SA treatment (Figure 6B). The ratio of ApoA1 to ApoB can reflect changes in the blood lipid levels more effectively, and in this study, its trend was consistent with ApoA1 (Figure 6C). In conclusion, SA has a certain effect on lowering the blood lipids and may have a positive effect on the prevention of vascular diseases, which may improve AD.

### 3.5. SA May Affect Liver Function in AD Mice

**Figure 7:**
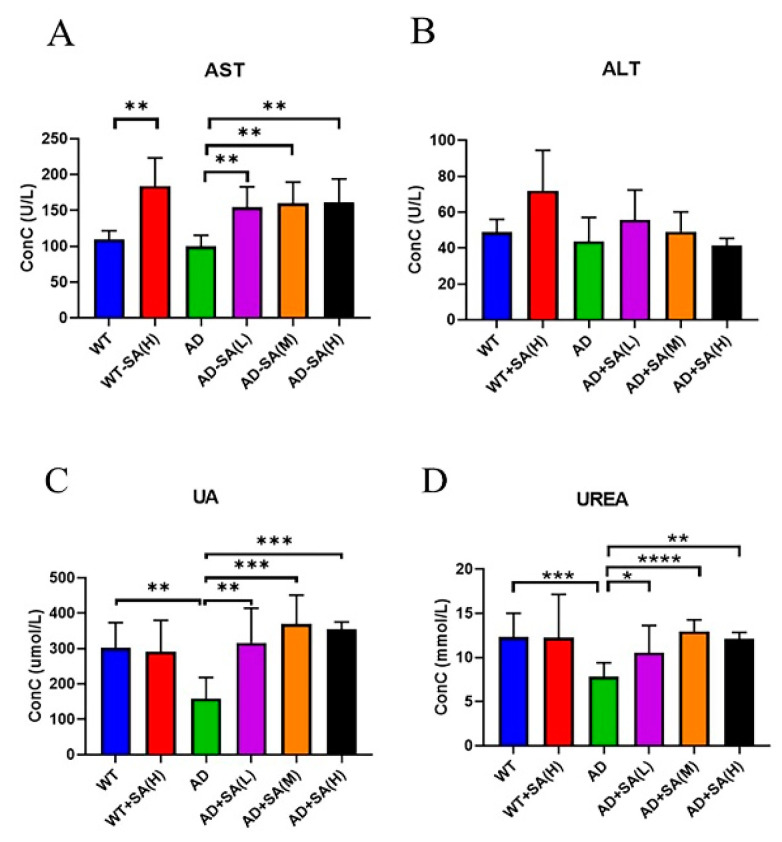
Effect of SA treatment on liver and kidney indicators in 2×Tg AD mice. (**A**) Aspartate transferase. (**B**) Alanine aminotransferase. (**C**) Uric acid. (**D**) Urea (* *p* < 0.05, ** *p* < 0.01, *** *p* < 0.001, and **** *p* < 0.0001). Student’s *t* test was used for statistical analysis. Error bars represent the mean ± SEM (*n* = 6).

Aspartate transferase (AST) and alanine aminotransferase (ALT) are used to evaluate liver function [20]. There was no difference between AD mice and WT mice, while feeding SA significantly increased AST (Figure 7A). No difference in ALT was found (Figure 7B). These two indicators denoted that SA may damage liver function.

As for kidney function, uric acid (UA) and urea are important. UA is the final product of purine metabolism, and a change in it can fully reflect the human body’s metabolic, immune, and other functions [21]. Urea is a product of human protein catabolism [22]. Excessive uric acid and urea involve liver and kidney damage, while an increase in UA in the normal range can hinder the onset of AD [23]. From the results, the UA and UREA of AD mice were significantly lower than that of WT mice, and SA treatments significantly increased the UA and UREA in AD mice, approaching the WT level (Figure 7C,D).

### 3.6. SA Has a Little Effect on Inositol, Glycine and Taurine

**Figure 8:**
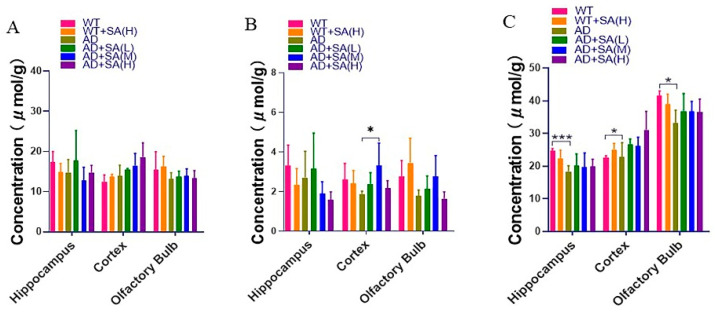
Effect of SA treatment on common small molecules in the brain in 2×Tg AD mice. (**A**) Myo-Inositol. (**B**) Glycine. (**C**) Taurine. Student’s *t* test was used for statistical analysis. Error bars represent the mean ± SEM (*n* = 4; * *p* < 0.05; *** *p* < 0.001).

Inositol, glycine, and taurine are the common small molecules in the brain, related to brain development and neurotransmission and so on [24,25,26]. From the results, the inositol concentration in different brain tissues was relatively stable, and SA has no significant effect on the inositol concentration in AD mice (Figure 8A). Glycine in the cortical of AD mice with a medium concentration SA treatment was significantly higher than that of AD mice, although there was no significant difference in other brain regions (Figure 8B). In the hippocampus, cortex, and olfactory bulb area, the taurine in AD mice was significantly lower than that of WT mice. After feeding different concentrations of SA, the taurine concentration increased but there was no significant difference (Figure 8C).

## 4. Discussion

AD is a progressive neurodegenerative disorder representing the most common cause of dementia in elderly people. The main clinical symptoms of AD patients are progressive memory loss and cognitive impairment [27]. Typical pathological features are the accumulation of a large number of Aβ plaques in brain tissue, nerve fiber tangles in brain cells, neuron loss, and so on, resulting in inflammation, oxidative stress, impairment in vasorelaxation, and progressive brain atrophy [1,11,12,28]. Many studies support the idea that SA can enhance brain development, cognition, and learning behavior of animals [7,29,30]. Exogenous free SA can cross the blood–brain barrier (BBB) and enter various tissues [5]. Therefore, improving AD through dietary intake of SA is undoubtedly a safe and effective way, and clinical application is also preferable. However, there are few studies on SA interfering with the treatment of AD. In this study, we employed an APP/PS1 transgenic mouse model that developed age-related accumulation of plaques and tangles in the brain, showing some symptoms related to AD, to test the hypothesis that SA could be effective in treating AD. The results showed that compared with AD mice, SA improved the spatial learning and memory ability and anxiety of mice, correlating with preventing the loss of neurons and with reducing the accumulation of some Aβ oligomers and the formation of neurofibrillary tangles. Moreover, it may ameliorate AD symptom by improving blood lipid function and preventing cardiovascular and cerebrovascular diseases, although slight damage to liver function occurred in AD mice treated with SA.

The water maze experiment is the most classic experiment to test the spatial learning and memory ability of mice [8]. The escape latency of the 5 days’ spatial training, swimming speed, time in target quadrant, and the number of crossing platform reflects their learning ability, movement ability, and spatial memory ability [9]. In this study, the escape latency of each group was shortened day by day, and even on the fifth day, AD mice treated with medium and high concentrations of SA were significantly shorter than AD mice. Although only the AD mice treated with a high concentration of SA stayed significantly longer than AD mice in the target quadrant, the platform crossing numbers of all the SA treatment at 24 h or 72 h was significantly higher than that of AD mice. These results demonstrated that SA significantly improved cognitive ability in AD mice, especially at medium and high concentrations.

The open-field experiment, which exploits the innate avoidance to open fields and exploration of new things by mice, evaluated the spontaneous activity and anxiety of mice [10]. In this study, the results showed that long-term feeding of SA increased the number of rearing and crossing grids of AD mice, indicating that the spontaneous activity and anxiety of AD mice were improved by SA treatment. Combining the results of the water maze experiment and open field experiment, it showed that long-term feeding of SA had a significant therapeutic effect on impaired cognitive ability and motor ability of AD mice, especially at the medium and high concentration.

As mentioned earlier, Aβ and hyperphosphorylated tau are two major toxic proteins of AD [11]. Aβ, which is mainly produced by the abnormal shearing of amyloid precursor of protein (APP) by β-amylase and γ-secretase, respectively, is the core of senile plaque (SP) and plays a key role in AD pathogenesis [12,31,32]. Tau originally promotes the assembly of microtubules and maintains the function of the cytoskeleton, while tau, which is over-phosphorylated, not only loses its original biological function but also dissociates from the microtubules and aggregates with each other, eventually forming a large number of NFT [14,33]. Previous studies have shown that Aβ has a strong toxic effect and induces the formation of hyperphosphorylated tau, which eventually leads to neuronal degeneration, dysfunction, and death [34,35]. In this study, using the brain section thioflavin staining and silver staining, it was observed that the number or area of beta amyloid in SA-fed AD mice was reduced in certain brain regions, most significantly in the medium concentration SA treatment, demonstrating the Aβ-clearance effect of SA. The number of NFT in AD mice also decreased with the increase of SA concentration. Neuronal damage and death are pathological features of AD, especially in brain learning and memory-related areas [28]. Neurons are closely related to Nissl bodies, brain learning and memory rely on neurons to conduct excitement, and the required proteins are synthesized by Nissl bodies [16]. Therefore, the number activity of Nissl bodies can indirectly reflect the function of neurons. In this study, the number of Nissl bodies in AD mice was significantly less than that in WT mice, but it increased after SA feeding, indicating that SA restored neuronal function to a certain extent. Therefore, SA reduced the neuron damage by reducing Aβ and inhibiting tau protein hyperphosphorylation, which improved the cognitive ability and mobility of AD mice, especially in the medium concentration treatment.

It is well known that obesity, hypertension, cardiovascular, and cerebrovascular diseases increase the risk of AD [36,37,38,39]. Lipoproteins are complexes of different lipids and proteins that act on the transport and clearance of lipids or lipid-related molecules in the circulation [17]. Lipoproteins also play a vital role in brain function, such as high-density lipoprotein (HDL), and its main protein component ApoA1 directly participate in the outflow of cholesterol in the brain [40,41]. Previous studies have shown that overexpression of human ApoA1 in the circulation can prevent learning and memory impairment in APP/PS1 transgenic mice, partly by reducing neuroinflammation and cerebral amyloid angiopathy [41]. In addition, previous research found that ApoB increased in AD plasma and serum, and over expression of ApoB in transgenic mice caused triggers apoptosis and neurodegeneration in the brain [42,43,44]. In this study, compared with WT mice, the ApoA1 levels significantly decreased and the ApoB significantly increased in AD mice, which is consistent with previous studies [41,42,43]. After feeding SA for 6 months, the levels of ApoA1 and ApoB were greatly improved, and which were significantly different from that of AD mice. Furthermore, the ratio of ApoA1/ApoB was consistent with ApoA1. Previous research has shown a higher ApoB/ApoA1 ratio indicates a higher risk of cognitive decline in the future [45].

From the results, SA has a certain effect on lowering blood lipids and may have a positive effect on the prevention of vascular diseases, which may improve AD.

ST and ALT are the most sensitive indicators of liver cell damage. AST and ALT increase significantly in large amounts in serum when liver cells are severely damaged [20]. In this study, both AD mice and WT mice fed with SA significantly increased the AST levels, but there was no difference in AST levels among the groups. This showed that SA may slightly damage liver function.

Among the possible markers of age-related cognitive decline, UA is controversial because it has antioxidant properties, but high UA increases the risk of gout and cardiovascular disease and affects kidney function [46]. Al-Khateeb, Althaher [23] found that serum UA in AD patients was significantly lower than in healthy controls. In this study, we also found that the UA of AD mice was significantly lower than that of WT mice, while a normal UA level was observed in the SA-treated AD mice. Thus, to some extent, it suggested that SA may improve the antioxidant ability in AD mice; further indicators about the antioxidant properties remain to be detected. However, whether UA reduction is the result or the cause of AD remains to be further studied. Urea is the end product of human protein metabolism. When the kidney is severely damaged, the urea content in the serum will increase [22]. In this study, although SA feeding increased urea in AD mice, there was no significant difference with WT mice. Combined with uric acid and urea, SA did not damaged the kidneys, but only reversed the UA and urea of AD mice to normal levels.

Inositol is closely related to signal transduction, and its imbalance will lead to neurological disorders [25]. Many studies have proved that inositol can improve the pathological characteristics of AD model animals and reverse cognitive impairment [47,48]. However, in this study, inositol was stably expressed in different groups. Glycine is a neurotransmitter with anti-inflammatory, signaling, and cellular immunity effects [26]. Inhibition of glycine transporter-1 (GlyT1) is expected to increase glycine, thereby reducing cognitive impairment in AD model animals [49]. We also found that the glycine in the cortex of AD mice after the medium concentration SA treatment was significantly higher than that of WT mice in this study, and it may be a good phenomenon. Taurine decreases in the mammalian brain with age, affecting memory ability [50]. In this study, the taurine in the hippocampus, cortex, and olfactory bulb area of AD mice was significantly lower than that of WT mice. After feeding different concentrations of SA, the taurine concentration increased but there was no significant difference, consistent with studies showing taurine’s therapeutic effect on AD [24,51,52].

## 5. Conclusions

(1) SA reduced the neuron damage by reducing Aβ and inhibiting tau protein hyperphosphorylation, which improved the cognitive ability and mobility of AD mice.

(2) SA has a certain effect on lowering blood lipids and may have a positive effect on the prevention of vascular diseases, which may improve AD.

(3) SA may slightly damage liver function.

## Data Availability

The data presented in this study are available in the main text, figures, tables.
